# Ischiopubic Ramus Resection as Treatment for Giant Cell Tumor of the Bone: Surgical Techniques in Two Clinical Cases

**DOI:** 10.7759/cureus.45661

**Published:** 2023-09-21

**Authors:** Diogo Sousa, Sérgio Pita, Vânia Oliveira, Pedro Cardoso

**Affiliations:** 1 Orthopaedics and Traumatology, Centro Hospitalar Trás-os-Montes e Alto Douro, Vila Real, PRT; 2 Orthopaedics and Traumatology, Centro Hospitalar Universitário do Porto, Porto, PRT; 3 Musculoskeletal Tumors Unit, Orthopaedics, Centro Hospitalar Universitário do Porto, Porto, PRT

**Keywords:** ischiopubic ramus resection, ischium, treatment outcome, surgical procedures, pelvic tumor, musculoskeletal oncology, orthopedics and trauma, giant cell tumor of bone

## Abstract

Giant cell tumors (GCTs) of the ischium are rare and often diagnosed at an advanced stage. In fact, there is no defined treatment algorithm to treat this lesion. We present two case reports of Campanacci's stage three ischiopubic GCT confirmed with biopsy. They were effectively treated with excision of the ischiopubic ramus, aggressive curettage, drilling, and phenolization at the margins. The surgery was performed in a gynecological position with an approach over the ischiopubic ramus. Both cases present no recurrence (two and 10-year follow-up), and neither has a significant impact on the quality of life. A thorough plan and surgical technique were essential for the success of this intervention.

## Introduction

Giant cell tumors (GCTs) of bone are locally aggressive benign neoplasms, although they can develop lung metastasis and suffer malignant transformation. They correspond to 5% of all primary bone tumors and 20% of benign tumors. Most cases are diagnosed between the second and fourth decades of life, with females being slightly more common. In 90% of cases, they appear in the epiphysis of long bones, close to the joint. About half of these tumors are found in the knee region (distal femur or proximal tibia) [1]. The Campanacci classification is the most used and characterizes them into three grades according to their radiological appearance: grade 1: inactive, grade 2: active, and grade 3: aggressive [2].

Pelvic GCTs are very rare, corresponding to only 1.5%-6.1% of all GCTs [3]. The classifications by Enneking and Dunham group them according to their location in the innominate bone. They are as follows: A: iliac wing (34.5%), B: peri-acetabular region (43.2%), and C: ischium and pubis (22.3%) [4,5]. Due to its rarity, its profound location, and its unspecific initial presentation, the diagnosis is usually late, and there is no defined treatment protocol.

Therapeutic options include radiotherapy, intralesional resection, and wide resection, with or without adjuvant treatments such as radiotherapy, angioembolization, denosumab, and bisphosphonates [6].

Pelvic GCTs present higher rates of complications and recurrence when compared to other sites [2]. The rates of complications described are 50% for radiotherapy, 14.3% for intralesional resection, and 46.4% for wide resection. The rates of recurrence described are 45% for radiotherapy, 33.3% for intralesional resection, and 2% for wide resection [5]. It should be noted that 70% of local recurrences occur within two years [7].

The best treatment should be adapted to the characteristics and anatomy of the lesion, namely the relationship with neighboring joints, as well as the patient's expectations. These lesions can reach considerable dimensions and extend into the acetabulum, leading to significant morbidity associated with the surgical treatment.

## Case presentation

**Figure 1:**
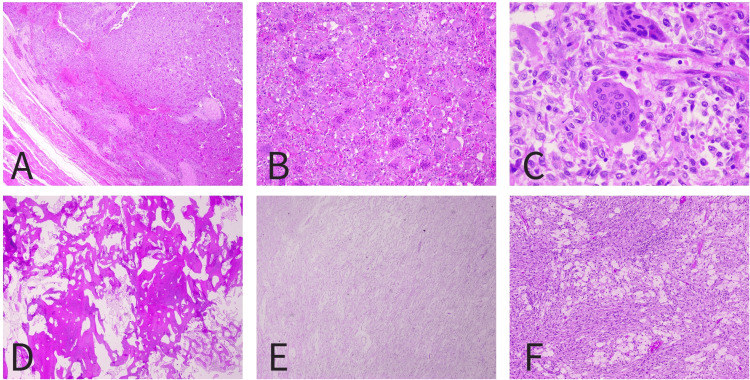
Histopathological images of the male patient's biopsy (A, B, and C) with the usual histology of a giant cell tumor of the bone, and of the female patient's biopsy (D, E, and F) after denosumab treatment (A) H&E 20x: The tumor invades the musculoadipose soft tissues with an expansive growth pattern; (B) H&E 200x: It consists of numerous giant multinucleated cells, some with numerous nuclei (giant cells of the osteoclast type), diffusely distributed throughout the tumor, and mononucleated cells, small and with little cytoplasm; (C) H&E 400x: The nuclei of mononuclear cells and multinucleated cells have similar characteristics, being euchromatic, slightly vesicular, and with slight polymorphism. (D) H&E 20x: The tumor is largely replaced by areas of osteosclerosis, characterized by the formation of thick and anastomosed bone trabeculae with fibrovascular intertrabecular spaces; (E) H&E 20x: Other areas are made up of low-cellularity myxofibrous stroma; (F) H&E 100x: Other areas still present spindle cell proliferation without atypia and with a storiform pattern, with frequent aggregates of foamy histiocytes. H&E: hematoxylin and eosin stain

Two patients, the first, a male in his 30s, and the second, a female in her 60s, presented with non-specific low back and sacroiliac pain and were diagnosed with Campanacci's stage three ischiopubic GCT, confirmed with biopsy (Figure 1).

The extension of the lesion to the posterior wall of the acetabulum in both cases precluded a wide resection. The first patient underwent preoperative angioembolization, and the second one completed one year of neoadjuvant denosumab (Figure 1). Excision of the ischiopubic ramus with aggressive curettage, drilling, and phenolization at the margins was performed. Additionally, the first patient received adjuvant radiotherapy in the two months following surgery.

Treatment

**Figure 2:**
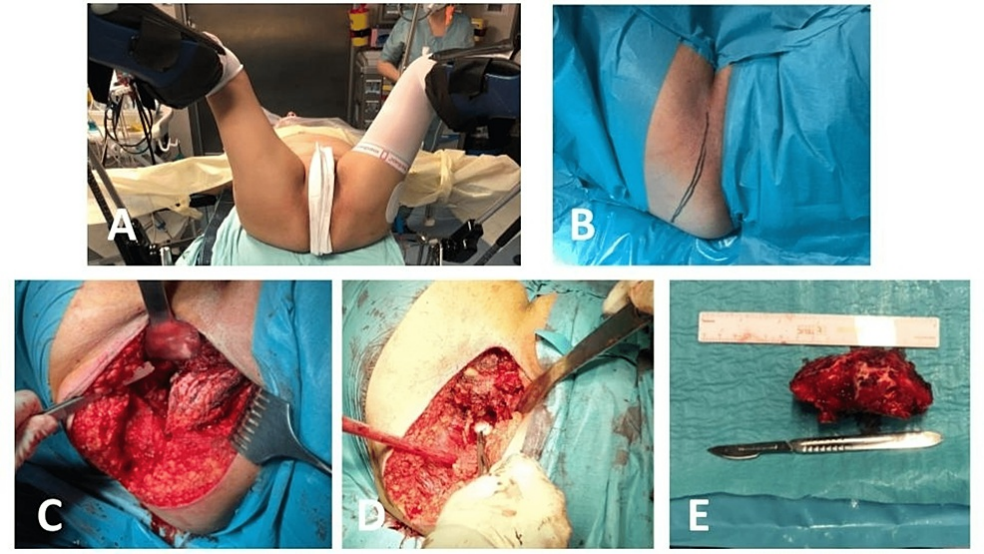
Intraoperative photographs of the second patient (A) patient supine in gynecological position; (B) planned longitudinal incision and exposure of the lesion after disinsertion of the muscles from the ischial tuberosity; (C) defect after resection of the ischiopubic ramus and resected lesion with ruler for scale; (D) phenolization of the margins; (E) resected lesion next to a ruler and scalpel for scale.

Surgeries were performed with the patient supine in a gynecological position (Figure 2A).

A longitudinal incision centered over the ischiopubic ramus was made (Figure 2B). After dissection of the muscles inserted in the ischial tuberosity, osteotomy of the ischiopubic ramus with intralesional margins adjacent to the acetabulum was performed, followed by aggressive curettage and phenolization of the margins (Figure 2C).

In the first case, the obturator nerve was sacrificed, and cement was applied to the acetabular margin to provide subchondral structural support.

Histological examination of the resected lesion confirmed the diagnosis of a giant cell tumor of the bone in both cases.

Outcomes and follow-up 

Both patients had uneventful wound healing.

**Figure 3:**
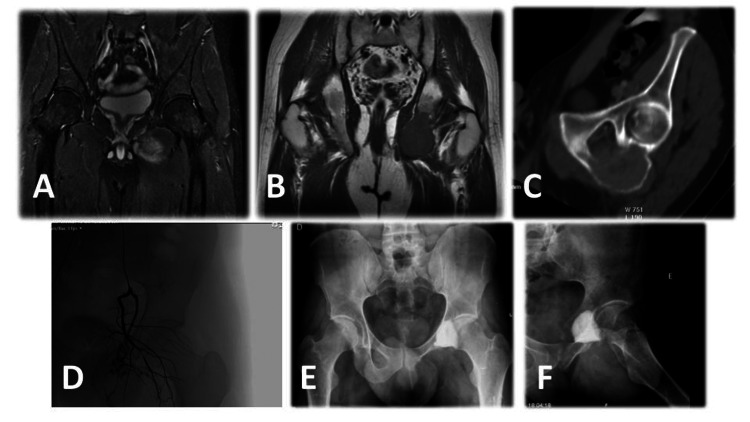
Radiological study of the first patient (A) preoperative MRI coronal STIR sequence; (B) preoperative coronal T1 weighted MRI; (C) preoperative CT scan; (D) preoperative angioembolization; (E) pelvis AP view; (F) frog-leg view postoperative radiographs showing ischiopubic ramus resection and subchondral bone support with cement. STIR: short tau inversion recovery; AP: anteroposterior

The first patient returned to work six months after surgery (as an intensive care nurse), however, he had paresthesias in the inner thigh and adductor weakness in the two months after surgery and developed chronic hip pain that was managed with oral pain medication. It has a mild impact on his daily activities. He had hip dysfunction and an osteoarthritis outcome score (HOOS) of 60.6% at 10 years of follow-up. He remains disease-free, without any signs of recurrence in the MRI (Figure 3).

**Figure 4:**
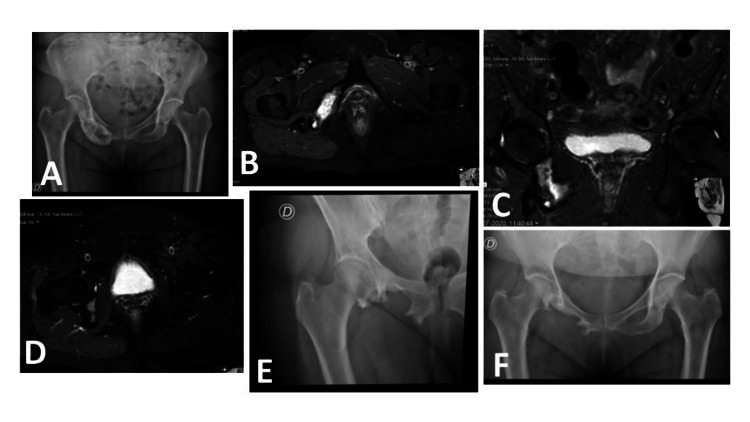
Radiological study of the second patient (A) preoperative pelvic AP view radiograph after treatment with denosumab; (B) preoperative T2 weighted MRI axial cut at the tumor level; (C) coronal; (D) axial cuts of the preoperative T2 weighted MRI showing the relationship of the lesion with the hip; (E) hip AP view; (F) pelvic AP view postoperative radiographs showing the ischiopubic ramus resection. AP: anteroposterior

The second patient is currently in the second year of follow-up and is asymptomatic after proper rehabilitation. She is disease-free from earlier breast cancer but presents slight peripheral neuropathy related to a previous systemic treatment (paclitaxel). She presents a HOOS of 86.9% and no signs of recurrence (Figure 4).

## Discussion

We report two clinical cases of ischiopubic ramus GCTs, locally aggressive with extension to the acetabular region, submitted to ischiopubic ramus resection and aggressive curettage at the margins.

The first patient, in his 30s (the typical age of presentation is between 20 and 40 years) [1], underwent selective arterial angioembolization and radiotherapy as adjuvant treatments. According to the literature, pre-surgical selective arterial embolization appears to be a safe procedure that may reduce the risk of local recurrence [8].

The second patient, in her 60s, underwent neoadjuvant denosumab for one year with tumor size reduction and better delimitation. The sclerotic borders still allowed proper curettage after the ramus resection. Denosumab is a human monoclonal antibody effective in the treatment of GCT of bone that cannot be surgically removed or when severe morbidity is expected with surgical resection [9]. The duration of treatment, side effects, long-term safety, and optimum indications for denosumab treatment are still unclear; hence, more studies are needed [10].

This specific anatomical location remains a challenge, and the adopted technique was successful. Surgical treatment of tumors affecting the acetabular region often results in functional impairment [8]. The first case presents mild hip pain with little repercussion on his quality of life, and both patients have no signs of recurrence.

Despite the challenge of the surgical approach, the complex anatomy of the pelvic region, and the local aggressiveness of GCT at the periacetabular area, we present two cases where the resection of the ischiopubic ramus and aggressive curettage of the margins were effective in treating and preventing recurrence with no significant morbidity to the patients.

Due to its rarity and the complexity of the local anatomy, there is no consensus on the treatment of GCTs of the ischiopubic ramus. In order to define a treatment algorithm or protocol, more studies are required, preferably multicentric, to have a significant sample and reliable results.

## Conclusions

Ischium GCTs are rare lesions with significant recurrence rates in the first two years, especially when the lesion is not completely excised. It has an unspecific and indolent presentation, which leads to late diagnosis and treatment.

An ischiopubic ramus resection is a good option for treating aggressive ischium GCTs. Extension of the tumor to the acetabulum may endanger the hip joint and preclude wide resection of the lesion, predicting a higher recurrence rate. Treatment must be individualized, depending on the stage of the disease, its location, and the expectations of the patient.
